# Efficient exosome delivery in refractory tissues assisted by ultrasound-targeted microbubble destruction

**DOI:** 10.1080/10717544.2018.1534898

**Published:** 2019-02-11

**Authors:** Wenqi Sun, Zhelong Li, Xueying Zhou, Guodong Yang, Lijun Yuan

**Affiliations:** a Department of Ultrasound Diagnostics, Tangdu Hospital Fourth Military Medical University, Xi’an, People’s Republic of China;; b The State Laboratory of Cancer Biology, Department of Biochemistry and Molecular Biology, Fourth Military Medical University, Xi’an, People’s Republic of China

**Keywords:** Targeted drug delivery, microbubbles, exosomes, efficiency, ultrasound-targeted microbubble destruction

## Abstract

Recently, exosomes have been emerged as promising drug delivery carriers, while certain tissues are intrinsically resistant to exosomes. Therapeutically improving the drug delivery efficiency in these tissues/organs would certainly broaden the potential application of exosomes in future. Ultrasound-targeted microbubble destruction (UTMD) is a promising technique for non-invasive, targeted drug delivery. In this study, we explore the possibility that UTMD assists exosome delivery in these intrinsically resistant tissues. Mice were subjected to tail vein injection of DiR-labeled exosomes together with/without UTMD of SonoVue^TM^, followed by *in vivo* and *ex vivo* tracking of the exosomes. As expected, heart, adipose tissue, and skeletal muscle were found reluctant to exosomes from different origins. Targeted destruction of the ultrasound microbubbles (SonoVue^TM^) in the heart and adipose tissue region significantly increased the exosome infiltration and endocytosis there, as revealed by fluorescence imaging and confocal laser scanning microscope (CLSM). UTMD treatment 1 h prior to exosome injection failed to facilitate the exosome endocytosis in the targeted region, indicating that the transient promoting effects of UTMD. Moreover, increases of UTMD (numerous pulses) did not linearly enhance the exosome delivery. Together, our study here has established a novel strategy for targeted delivery of exosomes in the reluctant tissues, by combining the advantages of ultrasound microbubbles and exosomes in drug delivery.

## Introduction

In the medicine field, gene therapy is the therapeutic delivery of nucleic acid into a patient's cells as a drug to treat disease. With the great advancement of RNAi, and gene editing with CRISPR/Cas9, gene therapy is now becoming a promising and possible strategy in the near future (Bak et al., 2018). One of the biggest hurdles of gene therapy is the challenge to non-invasively and locally deliver the gene drugs (Haussecker, 2014).

Exosomes, which are cell-derived vesicles of 30–150 nm in diameter, are emerging as a promising drug carrier (Contreras-Naranjo et al., 2017). In the physiological or pathological conditions, exosomes could transfer the bioactive molecules such as DNAs, RNAs, and proteins from the donor cells to the recipient cell, locally or distally (Kalluri, 2016). Moreover, the exosomes could be easily manipulated. The nucleic acids of interest could be either loaded by electroporation in the isolated exosomes or encapsulated during exosome biogenesis in the donor cells (Alvarez-Erviti et al., 2011; Barile & Vassalli, 2017). Exosomes have been reported to be resistant to the clearance by reticuloendothelial system and be able to cross multiple biological barrier (Alvarez-Erviti et al., 2011; EL Andaloussi et al., 2013). However, according to our preliminary data, exosomes remain resistant to certain tissues, with very low infiltration and endocytosed rate. Therapeutically increasing the exosomes delivery rate in these intrinsically refractory organs would certainly broaden the putative application of exosomes.

Recently, ultrasound-targeted microbubble destruction (UTMD) emerges as a novel means of tissue-specific gene delivery (Fujii et al., 2009, 2011). The approach delivers nucleic acids drug via the cavitation effect within the microvasculature of target tissues, which is especially of advantage for local delivery and the tissues with biological barriers (Hernot & Klibanov, 2008). However, the microbubbles should be loaded with the gene drugs prior to the UTMD. Currently, the ultrasound microbubbles are not efficient for gene loading and poor for long time preservation, restricting the potential application (Mullin et al., 2011; Tzu-Yin et al., 2013; Ma et al., 2017; Tamarov et al., 2017). In addition, the endocytosis efficiency is relatively low after the nucleic acids are released from the damaged microbubbles (Lee et al., 2012; Duan & Lam, 2013; Zhou et al., 2016).

In the current study, we explored the effects of targeted delivery of exosomes in the refractory tissues by using UTMD. With the assistance of the UTMD technique to regionally increase the vessel permeability, robust and localized delivery of the exosomes into the heart, adipose tissue, and muscle was achieved, shedding light on the gene therapy in these organs.

## Materials and methods

### Exosome isolation

Harvested tissues were cut into small pieces before further culture in the FBS free medium for another 24–36 h, followed by exosome isolation. For the isolation of exosomes, supernatants or the serum were centrifuged at 500 *g* for 10 min to remove cells and then at 10,000 *g* for 20 min to eliminate the residual cellular debris. The resulting supernatant was regularly filtered through 0.4 μm filters. The sample was used to precipitate exosomes with Exoquick-TC^TM^ kit. After that, exosomes were re-suspended in PBS or DMEM and stored at −80 °C. The morphology of isolated exosomes was analyzed by electron microscopy. Briefly, the exosomes were added onto the grid and stained with 2% uranyl acetate, followed by imaging with the transmission electron microscope (JEM-2000EX TEM, JEOL Ltd., Tokyo, Japan). Isolated exosomes were diluted to 500 ng/ml and subjected for size distribution analysis by Nanoplus.

### Animal housing

Male C56BL/6 mice (8–10 weeks old, 22–25 g) were used for *in vivo* analyzing the effects of ultrasound targeted microbubble destruction on exosome redistribution. Mice were maintained in a room with 12-h light-dark cycle and the temperature kept between 22 and 24 °C. All animal experiments were carried out under protocols approved by the Animal Care and Use Committee of Fourth Military Medical University.

### Ultrasound targeted microbubble destruction

SonoVue^TM^ microbubble (Bracco Imaging) was injected via tail vein at the dose of 100 µL or otherwise indicated. Ultrasound was generated by a 0.66 MHz low-power US instrument (Gift from Chongqing Medical University) with the probe area of 4.5 cm^2^. The nominal spatial peak-temporal average (SPTA) intensity varied from 0.22 to 1.80 W/cm^2^. The probe was adjusted with a gel interface so that the focus was positioned at the aimed tissues. For ultrasound irradiation, mice were anesthetized with 2% isoflurane, and 100 µL SonoVue^TM^ microbubble solution was infused into the tail vein slowly. Simultaneously, an ultrasound beam was delivered using the parameters as indicated.

### Exosome tracking in vivo and ex vivo

For *in vivo* tracking exosomes, purified exosomes were first labeled with fluorescent dye DiR/DiI at the final concentration of 10 µM (Invitrogen, Carlsbad, CA) before tail vein injection. Labeled exosomes were then collected by centrifugation after washed with PBS. Mice with/without ultrasound targeted microbubble destruction at indicated sites were additionally injected with labeled exosomes (100 µg per mouse, at 100 µL in volume) via tail vein before, after or at the same time of UTMD.

The mice were sacrificed at the indicated time after ultrasound irradiation and exosome injection. Different tissues from the mice that injected with DiR-labeled exosomes were harvested for fluorescence imaging. IVIS^®^ Lumina II *in vivo* imaging system was used for *in vivo* and *ex vivo* visualization of the exosomes as instructed. While different tissues from the mice that injected with DiI-labeled exosomes were harvested for sliced sections. For slice section staining, the specimens of different tissues were immediately harvested, embedded in optimal cutting temperature compound (OCT, Tissue-Tek, Torrance, CA). Tissue sections were fixed with 4% paraformaldehyde for 15 min and then stained with Hoechst (Invitrogen, Carlsbad, CA) for counterstaining of the cell nuclei. The whole process was conducted in dark. The fluorescence signal for the labeled exosomes and the blue nuclei were viewed by CLSM (ECLIPSE Ti, Nikon, Tokyo, Japan).

### Statistical analysis

All the data are expressed as mean ± SEM. Student’s *t* test was used for two group comparison, and one-way ANOVA was used for multiple comparisons by Tukey’s post hoc test (Graphpad Prism 7.0, GraphPad Software, La Jolla, CA). *p* values of <.05 indicate statistical significance.

## Results

### Exosomes preferentially distributed in reticuloendothelial system

**Figure 1.**
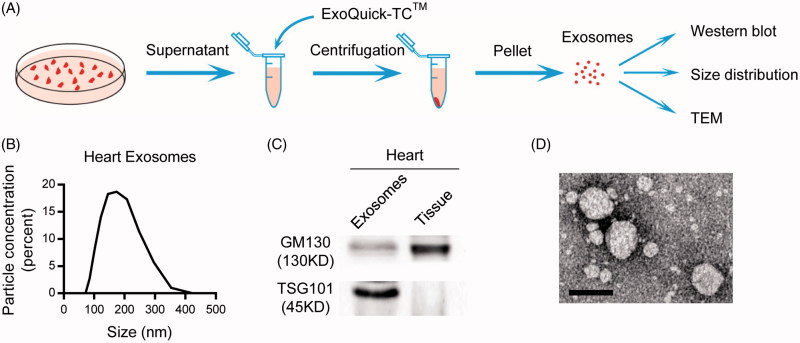
Isolation and characterization of exosomes. (A) Schematic representation of the exosomes isolation procedure. (B) Size distribution of the isolated exosomes. (C) Western blot analysis of exosomal markers in the isolated exosomes and the parental tissues. (D) Representative TEM (transmission electron microscope) image of the isolated exosomes (scale bar =100 nm).

Exosomes have been recognized as a promising drug carrier for targeted delivery. We thus explored the *in vivo* distribution of exosomes from different origins. For example, the heart tissues were cut into small pieces, cultured in the serum free medium and the secreted exosomes were isolated (Figure 1(A)). Size distribution analysis revealed that the size ranged from 60 to 200 nm in diameter (Figure 1(B)). The exosomal inclusive marker TSG101 was founded expressed in the exosomes, while the exclusive marker GM130 was absent (Figure 1(C)), confirming the identity of the isolated exosomes. TEM analysis of the morphology further confirmed the exosomes identity (Figure 1(D)).

**Figure 2.**
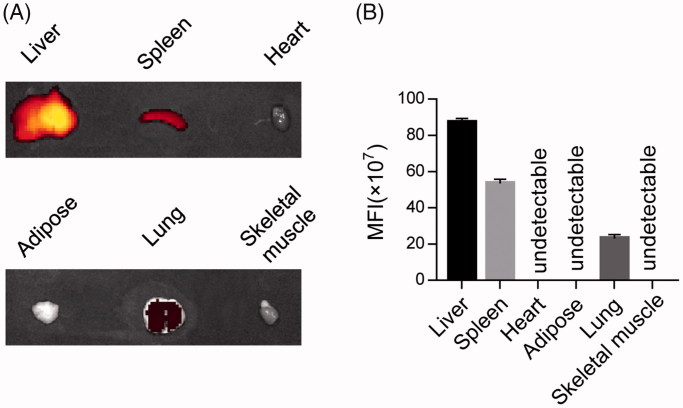
*In vivo* distribution of injected exosomes. (A) Fluorescence imaging distribution of the DiR labeled exosomes in different organs, including liver, spleen, heart, adipose, lung, skeletal muscle. Representative images of at least triplicate experiments. (B) Quantification of (A) (MFI: mean fluorescence intensity).

To track the *in vivo* distribution, isolated exosomes were labeled with DiR and injected via tail vein. Two hours later, the exosomes were found mainly localized in the liver, spleen, and lung, while rarely localized in the heart, adipose tissue, and skeletal muscles (Figure 2(A,B)), indicating that the exosomes mainly uptaken by the reticuloendothelial system *in vivo*. Similar results were observed in other tissue-derived exosomes.

### UTMD redistributes exosomes into targeted tissues

**Figure 3.**
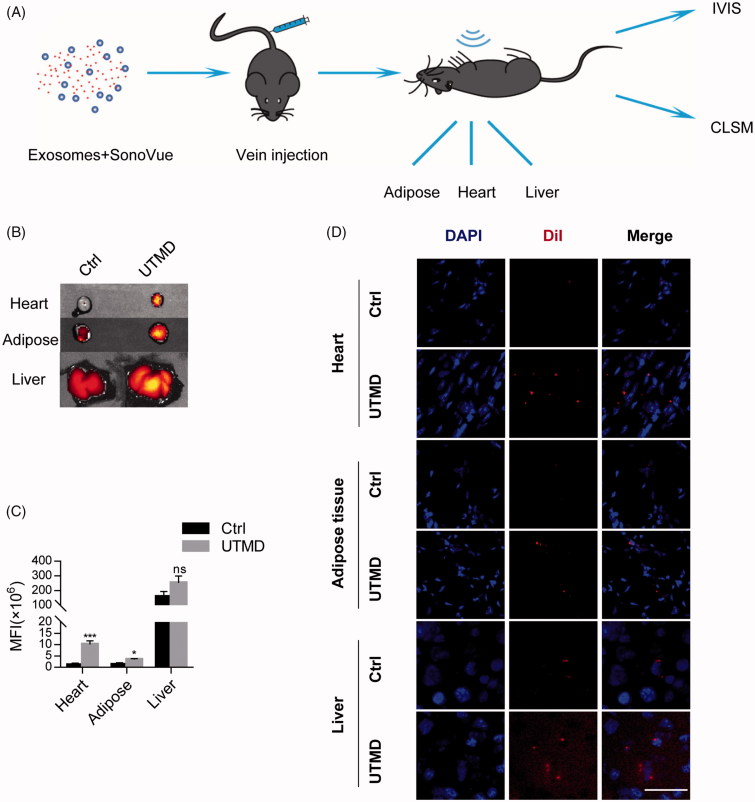
UTMD promotes the uptake of injected exosomes in the targeted tissues. (A) Schematic representation of the experimental procedure. Labeled exosomes, together with the SonoVue^TM^ microbubbles were injected via tail vein. UTMD was or was not induced in different tissues, including the heart, adipose tissue and liver. Distribution of the labeled exosomes was tracked by fluorescence imaging. (B) Fluorescence signal intensity in the tissues with or without UTMD. (C) Quantification of (B). (D) CSML image revealing the increased uptake of DiI-labeled exosomes in indicated tissues by UTMD. Nuclei were counterstained with Hoechst and scale bar =50 µm.

Enhancing localization of exosomes in the tissues like heart and adipose, is of fundamental importance for the treatment of associated diseases, especially when the prevalence of metabolic syndrome and cardiac disorders is considered. As UTMD could transiently destruct the microvasculature, we thus explored whether UTMD would facilitate exosomes infiltration in these naturally resistant organs. SonoVue^TM^ microbubbles were thus injected together with the exosomes and UTMD was induced specifically in the heart, adipose tissue, and liver (Figure 3(A)). As expected, *ex vivo* luminescence imaging revealed that UTMD significantly increased the infiltration of exosomes in these organs (Figure 3(B,C)). Moreover, UTMD also slightly increased the localization of exosomes in the liver (Figure 3(B,C)), although the robust localization already observed in the basic condition. CLSM analysis of the tissue sections further confirmed the enhanced exosomes distribution in the UTMD targeted tissues (Figure 3(D)).

### Effects of the UTMD duration and timing on the delivery efficiency

**Figure 4.**
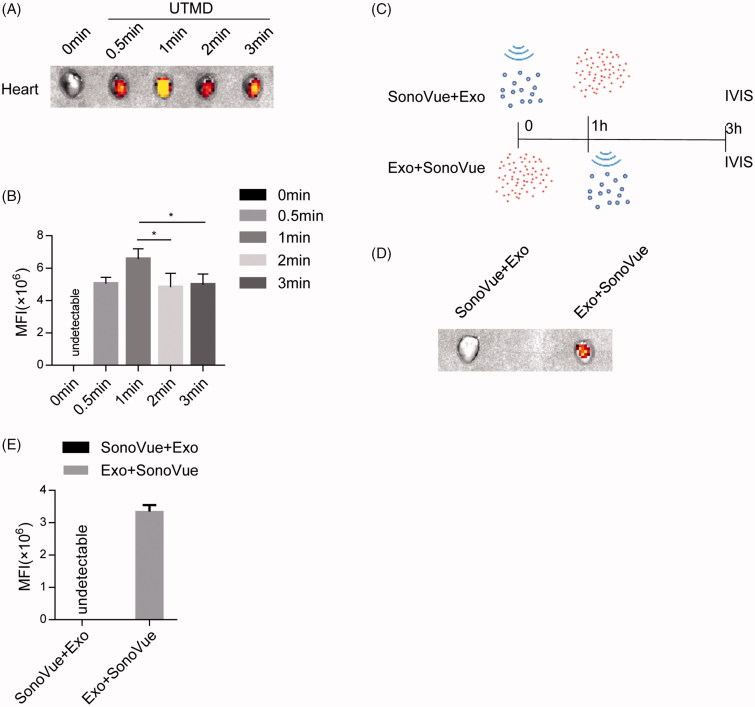
Effects of UTMD parameters on the uptake of injected exosomes in the targeted tissues. (A) Fluorescence imaging intensity reflecting the labeled exosomes distribution in the indicated tissues. Labeled exosomes with the SonoVue^TM^ microbubbles were injected via tail vein, and UTMD was induced by pulsed ultrasound with different time durations. (B) Quantification of (A). (C) Schematic representation of the exosomes injection and imaging procedure. Exosomes were either injected before or after UTMD, followed by tracking with imaging. (D) Biodistribution of the labeled exosomes in the indicated tissues. Labeled exosomes was injected 1 h before or after the SonoVue^TM^ microbubbles injection and destruction by UTMD. Simultaneous injection, followed by UTMD was also included. (E) Quantification of (D).

In view of above results, we next explored the effects of different UTMD duration on the exosome localization in the targeted heart tissue. Extension of the ultrasound radiation time from 0.5 min to 3 min did not increase the exosomes infiltration linearly as expected (Figure 4(A,B)), suggesting that the UTMD of 0.5 min or even less is enough for efficient delivery of exosomes to the targeted tissues.

In the following experiments, we also explored the effect of UTMD initiation time point on delivery of exosomes. Similar as the simultaneous injection, UTMD stimulation 1 h after exosomes injection remained to facilitate the delivery of exosomes in the heart (Figure 4(C–E)), suggesting that there remain amounts of free circulating exosomes 1 h after injection. In contrast, when UTMD was conducted 1 h before exosomes injection, rare exosomes infiltration was seen in the targeted heart (Figure 4(C–E)), indicating that the UTMD effects on facilitated exosomes delivery is transient and could not be maintained over one hour. The transient effects further strengthen the safety of UTMD in promoting exosomes targeted delivery in the refractory organs.

## Discussion

Our study here has revealed that targeted destruction of the microbubbles in the aimed region significantly facilitates the exosome endocytosis. To our knowledge, the study presented here for the first time solves the problem of delivering exosomes in the refractory tissues, such as heart and adipose tissues.

Targeted delivery of drugs, especially gene drugs, is essential for the optimal therapy (Rupaimoole & Slack, 2017; Rosenblum et al., 2018). The ideal drug delivery system should be safe and efficient (Naldini, 2015). Canonical drug carriers such as viruses, liposomes, and ultrasound microbubbles have been extensively studied (Sawant & Torchilin, 2012; Karimi et al., 2016). Take ultrasound microbubbles as example, although with several advantages as drug delivery vehicles, the non-uniform particle size distribution and difficulty in encapsulation of functional drugs (Cavalli et al., 2013; Jin et al., 2013) has limited their applications. In addition, the free diffusion of the drugs after ultrasound mediated destruction also compromises the drug delivery efficiency. On the other hand, accumulating evidence suggests that exosomes can deliver multiple cargos from the donor cells to the recipient cells in a natural pathway for genetic material transfer (Jiang & Gao, 2017). Natural exosomes from many tissues or cells have been confirmed to be therapeutically effective for many different diseases (EL Andaloussi et al., 2013). In addition, exosomes could be easily manipulated by surface functionalization and cargo encapsulation. For these natural characteristics, exosomes are being explored as drug and gene delivery vehicles. However, exosomes are preferentially and mainly endocytosed by liver, spleen, bone marrow (Smyth et al., 2015), although it is founded to cross the blood–brain barrier and maternal–placental barrier (Rufino-Ramos et al., 2017; Nair & Salomon, 2018). We here solve the problem by combining the advantages of the two techniques. Our study here revealed that the clinically available diagnostic microbubble SonoVue^TM^ significantly increased the endocytosis of exosomes in the refractory heart and adipose tissues. The study not only proposes a strategy for exosome delivery but also raises the possibility that diagnostic destruction of the microbubbles might also facilitate the endogenous exosomes endocytosis in the refractory tissues if UTMD occurs there.

As to the mechanism why UTMD facilitates the exosome delivery in the refractory tissues, we prefer the model that microbubbles destruction resultant cavitation effect is able to enhance cell membrane permeability, which in turn not only slows the local blood flow but also the uptake efficiency of recipient cells.

The primary limitation of this study is that we didn’t observe the effects of the system in treating myocardial disease models. Future work should evaluate the treatment effects in animal models by loading therapeutic drugs to native exosomes.

Together, our study here has established a novel strategy for targeted delivery of exosomes in the reluctant tissues, by combining the advantages of canonical ultrasound microbubbles and exosomes in drug delivery. Future studies refining the parameters for better efficacy and smaller side-effects would certainly forward the strategy in clinical settings.

## Conclusion

Exosome drug delivery is a promising strategy for target therapy. But their resistance to certain tissues, such as heart and adipose tissue, limits the use in these areas. In this study, we for the first time revealed that SonoVue^TM^ microbubble together with UTMD significantly increases the infiltration and endocytosis of exosomes these reluctant tissues. We also found that limit amount of UTMD is enough to promote exosome delivery and the promoting effects are transient, suggesting the safety of the proposed strategy. Overall, this study highlights the potent potential of UTMD in facilitating exosomes delivery in tissues like heart and adipose tissue, which is promising for cardiac diseases and metabolic syndrome management.
